# The tolerance to hypoxia is defined by a time-sensitive response of the gene regulatory network in sea urchin embryos

**DOI:** 10.1242/dev.195859

**Published:** 2021-04-16

**Authors:** Majed Layous, Lama Khalaily, Tsvia Gildor, Smadar Ben-Tabou de-Leon

**Affiliations:** Department of Marine Biology, Leon H. Charney School of Marine Sciences, University of Haifa, Haifa 31905, Israel

**Keywords:** Hypoxia, Gene regulatory networks, Sea urchin, Deoxygenation, Evolution and development, Skeletogenesis

## Abstract

Deoxygenation, the reduction of oxygen level in the oceans induced by global warming and anthropogenic disturbances, is a major threat to marine life. This change in oxygen level could be especially harmful to marine embryos that use endogenous hypoxia and redox gradients as morphogens during normal development. Here, we show that the tolerance to hypoxic conditions changes between different developmental stages of the sea urchin embryo, possibly due to the structure of the gene regulatory networks (GRNs). We demonstrate that during normal development, the bone morphogenetic protein (BMP) pathway restricts the activity of the vascular endothelial growth factor (VEGF) pathway to two lateral domains and this restriction controls proper skeletal patterning. Hypoxia applied during early development strongly perturbs the activity of Nodal and BMP pathways that affect the VEGF pathway, dorsal-ventral (DV) and skeletogenic patterning. These pathways are largely unaffected by hypoxia applied after DV-axis formation. We propose that the use of redox and hypoxia as morphogens makes the sea urchin embryo highly sensitive to environmental hypoxia during early development, but the GRN structure provides higher tolerance to hypoxia at later stages.

## Abstract (precis)

**Summary:** The use of hypoxia and redox gradients as morphogens makes sea urchin early development sensitive to environmental hypoxia. This sensitivity decreases later, possibly due to the gene regulatory network structure.

## INTRODUCTION

During the evolution of metazoans, animals were exposed to variations in oxygen levels and molecular mechanisms evolved to enable organisms to cope with hypoxic conditions (Semenza, 2012). However, it is still unclear whether these mechanisms are sufficient to protect marine organisms and, specifically, their embryos from the acute hypoxic conditions that become more common in the oceans (Altieri et al., 2017; Breitburg et al., 2018; Hughes et al., 2020). In the past 50 years the dissolved oxygen (O_2_) content of the global ocean has decreased by more than 2%, apparently due to warming that reduces oxygen solubility and increases biological consumption (Schmidtko et al., 2017). Recent studies indicate that oxygen loss in the oceans, termed deoxygenation, is more lethal to marine life than the direct effect of the rising temperatures or ocean acidification (Altieri et al., 2017; Breitburg et al., 2018; Hughes et al., 2020; Schmidtko et al., 2017; Vaquer-Sunyer and Duarte, 2008). The embryos of marine organisms could be highly sensitive to deoxygenation, especially embryos that use endogenous hypoxia and redox gradients as morphogens to guide the activation of gene regulatory networks (GRNs) during normal development (Chang et al., 2017; Coffman and Su, 2019; Cordeiro and Tanaka, 2020; Dunwoodie, 2009; Lendahl et al., 2009). Deciphering the structure and function of developmental GRNs that are activated by hypoxia and redox morphogens is key to understanding this fundamental regulatory mechanism as well as to assessing the expected effect of ocean deoxygenation on marine embryos.

The sea urchin embryo provides an attractive system to study the developmental GRNs that are driven by gradients of oxygen and redox levels and the effect of hypoxic conditions on these GRNs. Sea urchins are major grazers in shallow seas and coastal waters across the oceans (Pearse, 2006) and adult sea urchins were shown to be moderately sensitive to hypoxic conditions (Hughes et al., 2020; Low and Micheli, 2018; Suh et al., 2014; Vaquer-Sunyer and Duarte, 2008). The experimental advantages of sea urchin embryos and the role of the sea urchins in marine ecology make them a prominent model system for developmental and ecological studies (Pearse, 2006; Peter and Davidson, 2011; Sethi et al., 2012). The models of the gene regulatory networks that drive sea urchin early development are the state of the art in the field (Morgulis et al., 2019; Oliveri et al., 2008; Peter and Davidson, 2011). Importantly, the sea urchin GRNs use endogenous oxygen and redox gradients as developmental morphogens that drive the formation of the dorsal-ventral (DV) axis (Chang et al., 2017; Coffman et al., 2014; Suh et al., 2014).

**Fig. 1.**
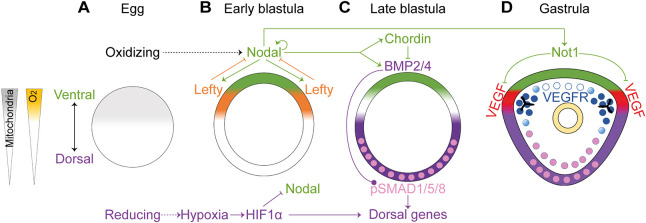
**The regulation of DV axis formation downstream of redox and oxygen gradients in the sea urchin embryo.** (A-D) Diagrams showing sea urchin DV and skeletal patterning in developing sea urchin embryos in normal conditions (based on Chang et al., 2017; Coffman et al., 2009; Coffman and Davidson, 2001; Coffman et al., 2004, 2014; Czihak, 1963; Duboc et al., 2004; Lapraz et al., 2009; Li et al., 2012). (A) The asymmetric distribution of mitochondria in the egg induces a redox gradient. (B) Regulatory interactions between *nodal*, *lefty* and HIF1α at the early blastula stage. (C) Nodal-mediated regulation of BMP signaling in the late blastula stage. (D) In the gastrula stage, Nodal activates the expression of Not1, which represses *VEGF* expression in the ventral ectoderm. Throughout the figure, the ventral side and Nodal expression domain are highlighted in green; the dorsal side and the domain of BMP activity are marked in purple. Nuclei that show pSMAD1/5/8 are highlighted in pink. *VEGF* expression is marked in red. *VEGFR* expression is marked in blue.

During early development of the sea urchin embryo, maternally induced oxygen and redox gradients initiate the localized activity of several signaling pathways that eventually control the patterning along the DV axis (Fig. 1; Chang et al., 2017; Coffman et al., 2014; Suh et al., 2014). In the eggs of the sea urchins, the mitochondria are concentrated at the future ventral side (Coffman et al., 2009, 2004), which leads to the formation of redox and oxygen gradients in the early embryos (Fig. 1A; Coffman et al., 2009, 2004). Apparently, the mitochondria produces reactive oxygen species (ROS) that activate redox-sensitive transcription factors that drive the expression of the Nodal ligand in the ventral ectoderm (Chang et al., 2017; Coffman et al., 2009, 2014; Nam et al., 2007; Range et al., 2007). Nodal reception drives the expression of the Nodal ligand and its antagonist Lefty, and the positive- and negative-feedback interactions between these two proteins define the boundaries of the ventral ectoderm (Fig. 1B; Duboc et al., 2008, 2004). Nodal activity drives the expression of the bone morphogenetic proteins (BMPs) BMP2/4, and its antagonist Chordin, forming an incoherent feedforward loop (Fig. 1C; Duboc et al., 2008, 2004; Lapraz et al., 2009; Saudemont et al., 2010). Chordin prevents the binding of BMP2/4 to their receptor at the ventral side, so BMP is received only at the dorsal side, where it activates gene expression through the phosphorylation of the transcription factor SMAD1/5/8 (Ben-Tabou de-Leon et al., 2013; Duboc et al., 2004; Lapraz et al., 2009) (Fig. 1C,D). Another early regulator of dorsal gene expression is the transcription factor hypoxia-inducible factor 1α (HIF1α), which is stabilized in the dorsal side of the sea urchin blastula, apparently downstream of the oxygen gradient (Fig. 1A,B; Ben-Tabou de-Leon et al., 2013; Chang et al., 2017). Thus, sea urchin embryos use the Nodal and BMP pathways, and HIF1α to generate their DV axis downstream of redox and oxygen gradients inherited from the sea urchin egg.

Growth in hypoxic conditions during early development, leads to radialization of sea urchin embryos with prominent effects on the larval skeleton (Agca et al., 2009; Coffman et al., 2004, 2014; Coluccio et al., 2011), while late hypoxia results with much milder phenotypes (Coluccio et al., 2011). The skeleton of the sea urchin larvae is made of two skeletal calcite rods, the spicules, that are formed within a tubular syncytial chord produced by the skeletogenic cells (Morgulis et al., 2019; Oliveri et al., 2008). When the embryos are grown in hypoxic conditions applied from fertilization and onwards to the blastula stage, the patterning along the DV axis and, specifically, skeletal patterning and elongation are severely disrupted (Coffman et al., 2004; Coluccio et al., 2011). Yet, when the embryos are exposed to hypoxic conditions from early blastula to mesenchyme blastula, their DV and skeletal patterning is largely unaffected (Coluccio et al., 2011). Little is known about the response of the GRN to these two distinct hypoxia treatments. Nodal expression has been shown to be spatially expanded under early hypoxia (Coffman et al., 2014) and the expansion of downstream markers of the ventral ectoderm is observed in this condition (Agca et al., 2009). However, the effect of hypoxia on the other upstream DV-patterning genes and on the skeletogenic GRN has not been investigated before, and the genetic response to late hypoxia has not been studied at all. Thus, early, but not late, hypoxia strongly disrupts DV and skeletal patterning, and the regulatory response to these two distinct conditions is poorly understood.

Sea urchin skeletogenesis depends on the vascular endothelial growth factor (VEGF) pathway, an essential regulator of vertebrates’ vascularization and of tubulogenesis in other phyla (Potente et al., 2011; Tettamanti et al., 2003; Tiozzo et al., 2008; Yoshida et al., 2010). The VEGF receptor (VEGFR) is expressed in the sea urchin skeletogenic cells together with five transcription factors whose homologs are essential for the vascularization of vertebrates (Adomako-Ankomah and Ettensohn, 2013; Duloquin et al., 2007; Morgulis et al., 2019; Sun and Ettensohn, 2014). This and other similarities between the sea urchin skeletogenic GRN and the vertebrate vascularization GRN suggest that these GRNs evolved from a common ancestral tubulogenesis GRN (Morgulis et al., 2019). The VEGF ligand is secreted from two lateral ectodermal domains located between the dorsal and the ventral ectoderm (Fig. 1D; Adomako-Ankomah and Ettensohn, 2013; Duloquin et al., 2007; Morgulis et al., 2019). VEGF expression is repressed in the ventral ectoderm by the transcription factor Not1, which is activated by Nodal signaling (Fig. 1D; Li et al., 2012). Yet, the regulatory links between BMP, HIF1α and VEGF signaling, and how VEGF and BMP pathways are affected by hypoxia are not known.

Overall, sea urchin DV axis formation and skeletogenesis are strongly affected by hypoxic conditions during early development, and are regulated by Nodal, BMP, VEGF and HIF1, downstream of maternal oxygen and redox gradients (Fig. 1). To understand the effect of exogenous hypoxia on sea urchin development, here we study the regulatory links between the sea urchin DV and skeletogenic GRNs during normal development and under hypoxia applied during either early or late development. We reveal that these two GRNs are strongly connected through the interactions between the BMP and VEGF pathways, and that the DV GRN is hypersensitive to hypoxia during early development but becomes relatively tolerant to low oxygen levels with developmental progression.

## RESULTS

### Sea urchin BMP2/4 control skeletal patterning and VEGF expression

**Fig. 2.**
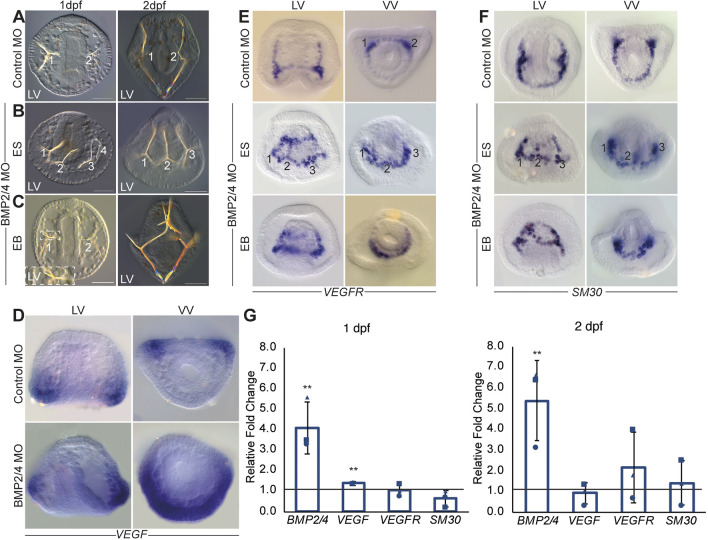
**BMP2/4** **control skeletal patterning and VEGF expression.** (A) Embryos injected with control MO show two normal spicules at 1 dpf (left, 110/110 of scored embryos show this phenotype) and 2 dpf (right, 56/56). (B,C) BMP2/4 MO-injected embryos show either ectopic spicules indicated by numbers (ES, 89/169 1 dpf, 120/135 2 dpf) or ectopic spicule branching (EB, 39/169 at 1 dpf, 15/135 at 2 dpf). Scale bars: 50 μm in A-C. (D) VEGF expression is localized in two lateral patches in the control embryo (top) and is strongly expanded in embryos injected with BMP2/4 MO at 1 dpf (bottom). (E,F) VEGFR and SM30 expression in control embryo (top) and in BMP2/4 morphants (middle and bottom) at 1 dpf. BMP2/4 MO leads to the expansion of the expression either into ectopic skeletal cell clusters indicated by numbers (ES) or to continuous expansion (EB). LV, lateral view; VV, ventral view. Phenotypes are based on *n*≥3 independent biological replicates and spatial expression was observed in two independent biological replicates where *n*≥30 embryos were scored in each condition. (G) Ratio between gene expression in BMP2/4 MO compared with control MO embryos at 1 dpf (left graph) and 2 dpf (right graph). Bars show averages and markers indicate individual measurements of three independent biological replicates. Line indicates a ratio of 1, i.e. the expression of the gene is unaffected by the perturbation. Error bars indicate s.d. Statistical significance was calculated using a one-tailed *z*-test (**P<0.01).

We first wanted to elucidate the links between BMP and VEGF signaling during normal sea urchin development. Previous studies had shown that the perturbation of BMP activity leads to the formation of ectopic spicules and to the dorsal expansion of the expression of the spicule matrix gene *SM30* (Duboc et al., 2010, 2004; Lapraz et al., 2009). However, the regulatory interactions that drive these phenotypes and, specifically, the regulatory links between the BMP and the VEGF pathways were unknown. To study these links, we knocked down BMP2/4 expression by the injection of translation morpholino oligonucleotides (MO) into the eggs of the Mediterranean sea urchin species *Paracentrotus lividus* (*P. lividus*, Fig. 2, see Materials and Methods for details). Embryos injected with BMP2/4 MO show two major skeletogenic phenotypes: the formation of ectopic spicules in addition to the normal two spicules (ES, Fig. 2B); and ectopic skeletal branching, where the basic structure of two spicules is still observed (EB, Fig. 2C). The expression level of *VEGF* is largely unchanged in BMP morphants (QPCR, Fig. 2G) but its spatial expression expands to one side of the ectoderm [detected by whole mount *in situ* hybridization (WMISH), Fig. 2D]. As BMP signaling induces dorsal specification (Duboc et al., 2004; Lapraz et al., 2009), *VEGF* expansion in BMP morphants is most likely to be the domain that would normally be specified as dorsal ectoderm. Hence, these data reveal that BMP activity represses *VEGF* expression and is essential for its localized expression in the two lateral ectodermal domains in normal embryos.

To further understand the regulatory links between the ectoderm and the skeletogenic GRNs, we studied the effect of BMP2/4 (KD) on the spatial expression of *VEGFR* and its target gene, SM30, at the gastrula stage (Duloquin et al., 2007; Morgulis et al., 2019). In control embryos, the expression of *VEGFR* is localized to the two skeletogenic cell clusters where the spicules first form (Fig. 2E) and the expression of *SM30* is noticeably enhanced in these clusters (Fig. 2F). BMP2/4 KD leads to two distinct expansion patterns of the expression of *VEGFR* and *SM30* (Fig. 2E,F). Some embryos show a continuous expansion of *SM30* and *VEGFR* expression that could drive the ectopic branching phenotype (EB in Fig. 2E,F). Similar expansion was observed for *SM30* expression in embryos where BMP activity was perturbed, and was interpreted as expansion to the dorsal skeletogenic cells (Duboc et al., 2010; Lapraz et al., 2009). However, in some embryos, *VEGFR* and *SM30* are expressed in three or four distinct cell clusters, which could be the cell clusters where ectopic spicules form in BMP2/4 KD (ES in Fig. 2E,F). The levels of *VEGFR* and *SM30* mRNA do not show significant change in BMP2/4 MO-treated samples at 1 and 2 days post fertilization (dpf, Fig. 2G). Overall, the expression of *VEGFR* and *SM30* expands in BMP KD, which could underlie the growth of ectopic spicules and ectopic spicule branches in this condition.

The expansion of *VEGFR* and *SM30* expression in BMP morphants is probably due to the combination of direct and indirect regulation of these genes by BMP signaling. *VEGFR* and *SM30* could be directly repressed by the BMP pathway through the phosphorylation of the transcription factor SMAD1/5/8 in the dorsal skeletogenic cells. Phosphorylated SMAD1/5/8 (pSMAD1/5/8) are indeed detected in the dorsal skeletogenic cells at the gastrula stage (Lapraz et al., 2009, 2006; Luo and Su, 2012) (Fig. 1D), where it activates the expression of *tbx2/3* and *gatac* (Duboc et al., 2010). The expression of *VEGFR* and *SM30* in BMP morphants could be also enhanced indirectly, through the expansion of *VEGF* expression in these embryos (Fig. 2D). Together, our results suggest that BMP2/4 signaling controls sea urchin skeletal patterning through the repression of *VEGF* expression in the dorsal ectoderm, and through the repression of *VEGFR* and *SM30* in the dorsal skeletogenic cells.

### HIF1α does not regulate skeletal patterning and VEGF expression in the sea urchin embryo

**Fig. 3.**
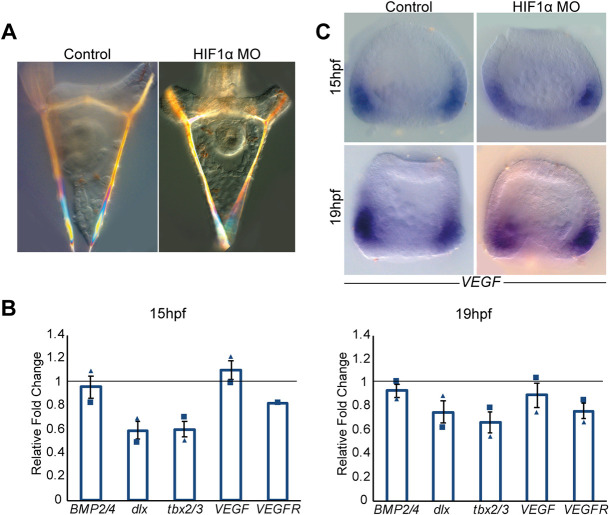
**Sea** **urchin HIF1α does not affect skeletal patterning and VEGF expression.** (A) Control (left) and HIF1α MO-injected embryos (right) show comparable skeletal structure at 2 dpf. (B) Ratio between gene expression in HIF1α MO compared with control MO embryos at 15 hpf (left graph) and 19 hpf (right graph). Bars show averages and markers indicate individual measurements of two independent biological replicates. Line indicates a ratio of 1, i.e. the expression of the gene is unaffected by the perturbation. Error bars indicate s.d. (C) *VEGF* expression is similar in embryos injected with control MO (left) and HIF1α MO (right) at 15 hpf (top) and 19 hpf (bottom). Spatial expression was observed in two independent biological replicates where *n*≥30 embryos were scored in each condition.

HIF1 is one of the most potent factors in the hypoxia pathway and, specifically, it activates *VEGF* expression during hypoxia-induced vascularization in vertebrates (Carmeliet, 2005; Pagès and Pouysségur, 2005). As the sea urchin HIF1α was shown to participate in early DV specification (Ben-Tabou de-Leon et al., 2013; Chang et al., 2017), we wanted to study the effect of the perturbation of this protein on sea urchin *VEGF* expression. In the sea urchin species *Strongylocentrotus purpuratus* (*S. purpuratus*), HIF1α KD reduced the early expression of the dorsal transcription factor Tbx2/3 and Dlx, reduced the extension of the dorsal apex, and mildly reduced the elongation of the dorsal skeletal rods (Ben-Tabou de-Leon et al., 2013; Chang et al., 2017). To study the effect of HIF1α perturbation on *VEGF* expression, we injected HIF1α translation MO into the eggs of the sea urchin *P. lividus* (Fig. 3). HIF1α KD did not result with distinct skeletogenic phenotypes, in agreement with its weak effect on *S. purpuratus* skeletogenesis (Chang et al., 2017) (Fig. 3A).

We tested the effect of HIF1α KD on gene expression level at two developmental time points: 15 h post-fertilization (hpf), which is equivalent to the developmental time where HIF1α activates its dorsal target genes in *S. purpuratus*; and 19 hpf, when the effect of HIF1α perturbation starts to decrease in *S. purpuratus* (Ben-Tabou de-Leon et al., 2013). HIF1α KD decreases the expression level of its known target genes, *Pl*-*tbx2/3* and *Pl*-*dlx*, with a stronger reduction at the earlier time point, similar to its effect in *S. purpuratus* (Ben-Tabou de-Leon et al., 2013), supporting the specificity of HIF1α MO (Fig. 3B). However, HIF1α KD does not affect *VEGF*, *VEGFR*, *BMP2/4* expression level at either time. Additionally, HIF1α KD does not affect the spatial expression of *VEGF* at either time points (Fig. 3C). Thus, our results indicate that HIF1α does not regulate VEGF expression in the sea urchin embryo, which is a noticeable difference from the prominent link between these two factors during vertebrate vascularization (Carmeliet, 2005; Pagès and Pouysségur, 2005).

### Rationale of acute early and late hypoxia treatments

Previous studies of the effect of hypoxia on *S. purpuratus* embryogenesis used different methods and various oxygen concentrations that lead to variable phenotypes, emphasizing the importance of experimentally defining the oxygen concentration throughout the hypoxia treatment (Agca et al., 2009; Chang et al., 2017; Coffman et al., 2009, 2004, 2014; Coluccio et al., 2011). Agca and colleagues decreased the oxygen concentration in the chamber where the embryos were cultured using a constant nitrogen (N_2_) flow, and observed a significant reduction in developmental rate when oxygen levels were less than 1% that became lethal at 0% oxygen (Agca et al., 2009). In these experiments, the dissolved oxygen level was not measured. In the first experiments by Coffman et al., the embryo culture was covered with a coverslip to reduce the gas exchange, but this experimental condition does not allow the measurement of the oxygen concentration (Coffman et al., 2004). Later, Coffman and colleagues used a transient flow of 100% N_2_ to reduce the dissolved oxygen level below 1 ppm (Coluccio et al., 2011) and, specifically, to 0.2 ppm (Coffman et al., 2014), and then sealed the chamber for the duration of the hypoxic treatment. In our system, we find it hard to completely seal the hypoxia chamber, yet growth in a constant flow of 100% N_2_ severely damaged the development of *P. lividus* embryos, similar to the reported lethal effect in *S. purpuratus* (Agca et al., 2009). We therefore sought to study the effect of transient acute hypoxia on *P. lividus* skeletogenesis and gene expression under controlled oxygen levels where the DV-axis is distorted but most of the embryos survive; such levels are relevant to environmental hypoxia.

The sensitivity to hypoxia changes significantly between different species and for adult sea urchin the reported sub-lethal threshold for hypoxia is 1.22 mg/l dissolved O_2_ (sub-lethal threshold means that the animals survive this stress but their growth, reproduction and physiology are damaged; Vaquer-Sunyer and Duarte, 2008). Water-quality surveys on sites where a massive mortality event occurred, detected levels of 0.5 mg/l O_2_ and below in the seabed at a depth of 10 m or deeper (Altieri et al., 2017). We therefore studied the effect of growth in 0.4-0.5 mg/l dissolved O_2_, which is a severe environmental hypoxic condition and is equivalent to 0.4-0.5 ppm (using a constant flow of 99.5% N_2_ and 0.5% O_2_, see Materials and Methods for experimental details). Embryos were cultured at 18°C, which is the typical temperature for the upper water column in the Mediterranean sea (Mavropoulou et al., 2020) and is higher than the culture temperature of *S. purpuratus* embryos (14-15°C; e.g. Agca et al., 2009).

**Fig. 4.**
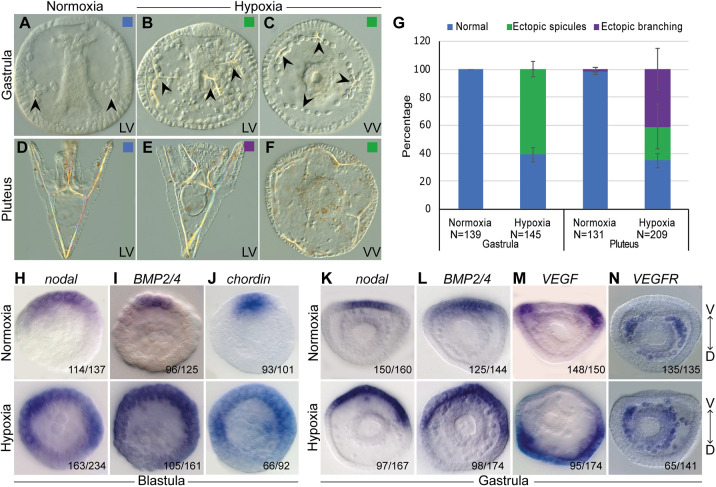
**Growth in hypoxic** **conditions leads to skeletal defects and perturbs the expression of DV and skeletal patterning genes.** (A-C) Representative images of embryos at gastrula stage. (A) Embryo grown in normoxic conditions shows normal development of two spicules (arrowheads). (B,C) Embryos grown in hypoxic conditions show ectopic spicules (arrowheads). (D-F) Representative images of embryos at pluteus stage. (D) Embryo grown in normoxic conditions shows a normal skeleton. (E) Embryo grown in hypoxic condition shows a normal DV axis and ectopic spicule branches. (F) Radialized embryo grown in hypoxic conditions that displays multiple ectopic spicules. LV, lateral view; VV, ventral view. (G) Quantification of skeletogenic phenotypes at gastrula stage and pluteus stage. Color code is indicated in the representative images. Error bars indicates s.d. of three independent biological replicates. (H-J) Spatial expression of *nodal*, *BMP2/4* and *chordin* genes in normoxic (top) and hypoxic (bottom) embryos at blastula stage. (K-N) Spatial expression of *nodal*, *BMP2*, *BMP4*, *VEGF* and *VEGFR* genes in normoxic (top) and hypoxic embryos (bottom) at the gastrula stage. Embryos are presented in ventral view and the axis is labeled ventral (V) to dorsal (D). Throughout H-N, the numbers at the bottom right indicate the number of embryos that show this expression pattern out of all embryos scored, based on three independent biological replicates.

**Fig. 5.**
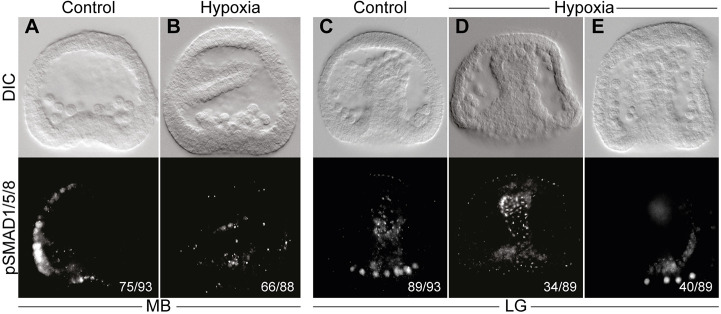
**BMP activity** **is reduced in hypoxic conditions.** (A,B) Nuclear pSMAD1/5/8 patterning in normoxic and hypoxic conditions at mesenchyme blastula (MB) stage. In normoxic conditions, pSMAD1/5/8 staining is detected in the dorsal ectoderm (A), while in hypoxic embryos the signal is completely abolished (B). (C-E) pSMAD1/5/8 staining in normoxic versus hypoxic embryos at late gastrula (LG) stage. pSMAD1/5/8 is detected in the nuclei of the dorsal skeletogenic cells of normoxic embryos (C), while in hypoxic conditions the signal is either not detectable (D) or strongly reduced (E). DIC images of the embryos are presented in the upper row of each panel; immunostaining of pSMAD1/5/8 of the embryos are presented in the lower row. All embryos are presented in lateral view (LV). The numbers shown on the bottom right of each figure indicate the number of embryos that show this expression pattern out of all embryos scored, based on three independent biological replicates.

**Fig. 6.**
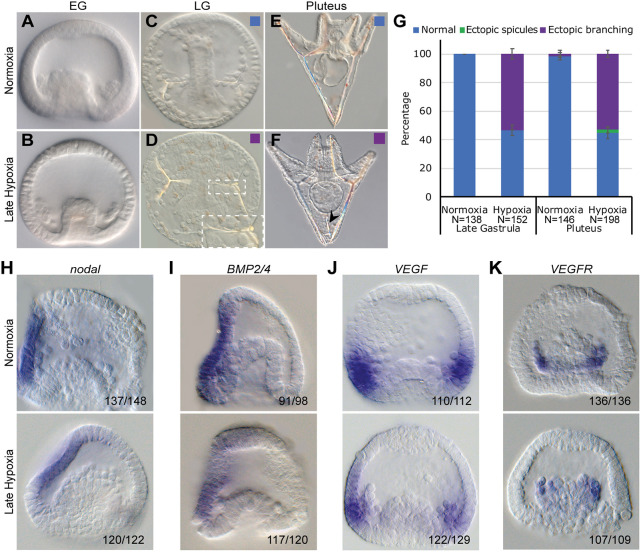
**Late hypoxia affects skeletal structure but not skeletal patterning.** (A-F) Representative images of live embryos: normoxic embryos are presented in the upper row and equivalently staged hypoxic embryos are on the bottom. (A,B) Embryos at early gastrula stage show similar morphology in normoxia and hypoxia. (C,D) A hypoxic embryo at late gastrula stage shows two spicules with ectopic spicule branching (D) that are not observed in the normoxic embryo (C). Inset shows the outlined area at higher magnification. (E,F) Embryos at pluteus stage. Arrowhead in F indicates an abnormal spicule growing in the hypoxic embryo. (G) Quantification of late hypoxia experiment over three biological replicates. Color code is indicated in the representative images. Error bars indicates s.d. of three independent biological repeats. (H-K) WMISH results of *nodal*, *BMP2/4*, *VEGF* and *VEGFR* at early gastrula stage. A normoxic embryo is presented at the top and a hypoxic embryo is at the bottom of each panel. On the bottom right of each figure, the number of embryos that show this expression pattern out of all embryos scored is provided, based on three independent biological replicates.

We specifically wanted to study the regulatory response to early and late hypoxia treatments (Coffman et al., 2014; Coluccio et al., 2011). Early blastula occurs in *P. lividus* embryos under normal conditions at about 10 hpf (Duboc et al., 2004; Lapraz et al., 2009), but when the embryos are grown in hypoxic conditions their development is slower and they reach this stage at 16 hpf. We therefore studied the effect of growth in hypoxic conditions (0.4-0.5 mg/l O_2_) for 16 h, from fertilization and onwards (early hypoxia, Figs 4–5), and from early blastula stage and onwards (late hypoxia, 10-26 hpf, Fig. 6, see Materials and Methods for the exact protocols). Our studies reveal significant differences in regulatory gene expression between these two treatments that can explain the distinct morphological and skeletogenic phenotypes.

### Early hypoxia distorts skeletal patterning and expands ventral and skeletal gene expression

Embryos grown for 16 hpf in hypoxic conditions that were applied immediately from fertilization and onwards (early hypoxia) are viable and develop into a normal-looking blastula, but show severe DV axis disruption and skeletogenic defects from the gastrula stage and onwards (Fig. 4A-G). This is in agreement with previous works on *S. purpuratus* and indicates that the effect of hypoxic conditions is not species specific (Agca et al., 2009; Chang et al., 2017; Coffman et al., 2009, 2004, 2014; Coluccio et al., 2011). At gastrula stage, most of the embryos grown in early hypoxia show an irregular skeleton with several ectopic spicules (61%, Fig. 4B,C,G). At pluteus stage, the embryos show partial recovery and display two major skeletogenic phenotypes: a strong phenotype in which the skeleton is radialized, the DV axis is disrupted and multiple ectopic spicules are observed (24%, Fig. 4F,G); and a weaker phenotype in which the DV axis seems normal but the skeleton shows ectopic spicule branching (41%, Fig. 4E,G). The rest of the embryos developed normally. The skeletogenic phenotypes indicate that hypoxic conditions can strongly affect skeletal patterning, probably through changes in skeletogenic gene expression.

Next, we investigated the effect of hypoxia on the expression of the DV patterning genes *nodal*, *BMP2/4* and *chordin* at blastula and gastrula stages in *P. lividus*. Growth in hypoxic conditions significantly expands *nodal* spatial expression throughout the ectoderm at blastula stage, compared with the ventral localized expression of this gene in normal development (Fig. 4H), in agreement with previous studies in *S. purpuratus* (Coffman et al., 2014). The spatial expression of *BMP2/4* and *chordin* show similar expansion at this time, as expected for downstream target genes of Nodal signaling (Fig. 4I,J). At early gastrula stage, the expression of *nodal*, *BMP2/4* is expanded in embryos grown in hypoxic conditions compared with the expression of these genes in embryos grown in normoxic conditions (Fig. 4K,L). However, the expansion at gastrula stage is not throughout the ectoderm as in the blastula stage, but seems more localized to about a half of the ectoderm, in agreement with the partial phenotypic recovery at the pluteus stage (Fig. 4G).

These results suggest that hypoxia leads to the expansion of the ventral ectoderm and probably to the decrease in the dorsal ectoderm domain, which may affect the expression of key skeletogenic regulators, such as *VEGF* and *VEGFR*. Indeed, growth in hypoxic conditions shifts and expands the spatial expression of *VEGF* to one side of the ectoderm, which is most likely the dorsal ectoderm (Fig. 4M). In addition, the expression of *VEGFR* expands beyond the two lateral skeletogenic cell clusters in which it is normally localized (Fig. 4N). Furthermore, the *VEGFR*-expressing cells demonstrate the perturbed migration of the skeletogenic cells in hypoxic embryos. This phenotype could be due to the expanded expression of the VEGF ligand that directs the migration of the skeletogenic cells in normal embryos. In summary, growth in hypoxic conditions perturbs the spatial organization of the skeletogenic cells and expands the ectodermal expression of *Nodal,* *BMP2/4*, *chordin* and *VEGF*, and the skeletogenic expression of *VEGFR.*

### Early hypoxia reduces BMP activity which explains *VEGF* and *VEGFR* expansion

The expansion of the ventral side in hypoxic conditions suggests that BMP activity at the dorsal side might be reduced, and the reduction of the repressing BMP activity could explain *VEGF* and *VEGFR* expansion to the dorsal side. To test this hypothesis and monitor BMP activity in normal versus hypoxic conditions, we performed immunostaining against pSMAD1/5/8. We studied pSMAD1/5/8 signal at two different developmental stages: mesenchyme blastula, when BMP activity is localized at the dorsal ectoderm (Fig. 5A); and late gastrula, when BMP activity is localized at the dorsal skeletogenic cells (Fig. 5C; Lapraz et al., 2009). Hypoxic conditions completely abolish pSMAD1/5/8 signal from the nuclei of the dorsal ectodermal cells at mesenchyme blastula stage (Fig. 5B). At late gastrula stage, hypoxic conditions eliminate the pSMAD1/5/8 signal from the dorsal skeletogenic cells (Fig. 5D), or strongly reduce it (Fig. 5E). These results indicate that, despite *BMP2/4* expansion in hypoxic embryos, its activity is reduced during hypoxia. The reduced activity can be explained by the expansion of a BMP antagonist, Chordin, during hypoxic conditions (Fig. 4J). Together, these results show that BMP activity in the dorsal ectoderm and in the dorsal skeletogenic cells is reduced in hypoxic conditions. Apparently, the reduction of BMP activity removes the repression of *VEGF* and *VEGFR* at the dorsal embryonic domains, and leads to their expansion to this domain and to the disruption of skeletal patterning.

### Late hypoxia mildly affects skeletogenesis and does not affect DV and skeletal regulatory genes

Our studies show that early hypoxia strongly affects the spatial activity of the main regulators of DV axis formation, Nodal, BMP2/4, and the perturbation of these factors affects skeletal patterning and *VEGF*, *VEGFR* and *SM30* expression. Next, we wanted to investigate the effect of late hypoxic conditions on regulatory gene expression. Thus, we studied the skeletogenic phenotypes and gene expression in hypoxia applied between 10 hpf and 26 hpf, i.e. starting from the early blastula stage. Embryos grown in late hypoxia showed delayed development and at 26 hpf were equivalent to early gastrula stage in normoxic embryos (Fig. 6A,B). At late gastrula and pluteus stages, almost all the embryos grown in late hypoxia show normal skeletal patterning with the two spicules correctly positioned at the two lateral sides (Fig. 6C-G), in agreement with previous studies in *S. purpuratus* (Coluccio et al., 2011). More than half of the embryos grown in late hypoxia developed ectopic skeletal branching in these two stages, and at pluteus stage, about 2% of the embryos show radialized skeleton with ectopic spicules. Overall, late hypoxia induces skeletal defects, such as ectopic branching, but it hardly affects skeletal patterning.

We next studied the effect of late hypoxic conditions on the expression of the key regulatory genes investigated above. Late hypoxia treatment does not affect the spatial expression of *nodal* (Fig. 6H), in agreement with the normal formation of the DV axis and normal skeletal patterning in this condition. Furthermore, late hypoxia does not affect the spatial expression pattern of *BMP2/4*, *VEGF* and *VEGFR* genes, so these genes are probably not the mediators of the observed mild skeletal defects (Fig. 6I-K). Thus, hypoxia applied from the early blastula stage and until early gastrula does not affect the expression of the upstream DV patterning and skeletogenesis regulators, *nodal*, *BMP2/4*, *VEGF* and *VEGFR*, which explains the overall normal morphology of the embryos in this condition.

## DISCUSSION

**Fig. 7.**
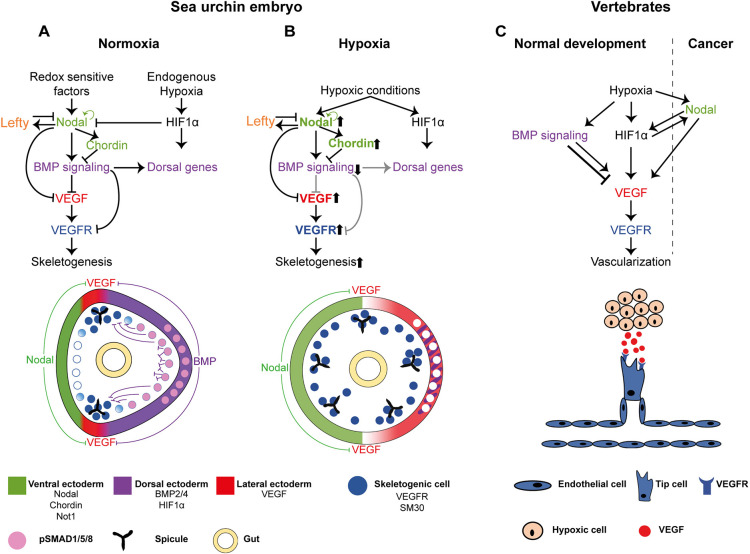
**The interactions between the DV and skeletogenic GRNs, the response to early hypoxia and the similarities to the regulation of vertebrate vascularization.** (A,B) Diagrams showing our proposed model for skeletal patterning in normal conditions (A) and hypoxic conditions (B). Color codes are indicated at the bottom of the figure. (A) The regulatory interactions between Nodal, BMP, HIF1α and VEGF signaling during normal development. BMP represses *VEGF*, *VEGFR* and *SM30* expression in the dorsal side, and HIF1α does not regulate VEGF expression in the sea urchin embryo. (B) The modification of the regulatory states in hypoxic conditions applied at early development revealed in this work. Early hypoxia expands *nodal* expression and reduces BMP activity and the dorsal ectoderm. The reduction of BMP activity leads to an expansion of *VEGF*, *VEGFR* and *SM30* expression in the dorsal side and to growth of ectopic skeletal centers. Upward arrows near a gene name indicate enhanced activity; downward arrows indicate reduced activity. Gray regulatory links indicate inactive connections under hypoxic conditions. (C) Diagram showing the relevant regulatory interactions during vertebrate vascularization in normal development and in cancer; see text for explanations.

GRNs are the genomically encoded programs that control embryonic development, but the environmental conditions in which these GRNs operate can significantly affect their outcome (Beldade et al., 2011; Smith et al., 2018). Particularly, the use of hypoxia and redox gradients to control developmental processes in various phyla, might make the embryos more sensitive to low oxygen levels that are becoming more common in the ocean (Compernolle et al., 2003; Cordeiro and Tanaka, 2020; Dunwoodie, 2009; Semenza, 2012). The structure of the developmental GRN defines its function during environmental hypoxia and underlies the response and resilience to hypoxia during embryogenesis. Here, we have studied the regulatory links and response to transient acute hypoxia of the GRNs that control DV patterning and skeletogenesis in the sea urchin embryo. Our results, together with previous studies, allow us to present a GRN model that provides a causal explanation for the skeletal radialization in early hypoxia, and the normal skeletal patterning in late hypoxia (Fig. 7A,B). Below, we discuss our main findings and their possible implications.

Our studies reveal the regulatory interactions between the DV and skeletogenic GRNs that underlie skeletal patterning in the sea urchin embryo. Previous studies had shown that *VEGF* expression is restricted from the ventral ectoderm by the Nodal target Not1 (Li et al., 2012); however, the mechanism that excludes *VEGF* expression from the dorsal ectoderm was not known. Here, we show that BMP activity restricts *VEGF* expression into two lateral ectodermal domains, and confines *VEGFR* and *SM30* expression into the two ventro-lateral skeletogenic clusters (Figs 2 and 7A). This restriction is necessary for spicule initiation to occur only in the ventro-lateral skeletogenic clusters. We also show that HIF1α, a key activator of VEGF in vertebrate vascularization (Dunwoodie, 2009; Pagès and Pouysségur, 2005), does not regulate VEGF signaling during early sea urchin development (Figs 3 and 7A). Apparently, the regulatory function of this factor in normal sea urchin development is limited to shaping the *nodal* expression domain in the early blastula (Chang et al., 2017) and to activating early dorsal gene expression (Ben-Tabou de-Leon et al., 2013). Thus, BMP signaling restricts VEGF activity to the ventro-lateral skeletogenic clusters and this restriction is required for the exclusion of spicule formation outside these clusters in normal sea urchin embryos (Fig. 7A).

Early hypoxia in sea urchin embryos strongly distorts the spatial expression of DV and skeletogenic patterning genes, which leads to the formation of ectopic spicules and embryo radialization (Figs 4,5 and 7B). Previous studies have shown that hypoxic embryos are ventralized (Agca et al., 2009) and that *nodal* expression expands in hypoxic conditions (Coffman et al., 2014); however, how hypoxia affects the DV and skeletogenic GRNs, which are downstream of Nodal signaling, was unclear. Here, we reveal the cascade of regulatory interactions that underlie embryo ventralization and the formation of ectopic spicules. Early hypoxia leads to the expansion of *nodal* to the dorsal side, which leads to the expansion of its targets, *BMP2/4* and *chordin* in this condition (Figs 4H-L and 7B). The activity of BMP signaling is significantly reduced in both the dorsal ectoderm and dorsal skeletogenic cells, as evident from pSMAD1/5/8 staining (Fig. 5). This reduction is probably due to the expansion of the expression of the BMP antagonist *chordin* into the dorsal side, which blocks BMP activity in early hypoxia embryos (Figs 4J and 7B). The expansion of Nodal activity excludes *VEGF* expression from the broader ventral domain, apparently due to *VEGF* repression by the Nodal target Not1 (Fig. 7B; Li et al., 2012). The reduction of BMP activity drives the expansion of *VEGF* and *VEGFR* expression, probably to the dorsal ectoderm and dorsal skeletogenic cells, respectively (Figs 4M,N and 7B). The expansion of VEGF activity explains the formation of ectopic spicules in early hypoxic condition (Fig. 7B). Hence, early hypoxia expands *nodal* expression, which reduces BMP activity and, specifically, removes the dorsal repression of VEGF signaling, which leads to the formation of ectopic spicules.

In striking contrast to the strong effect of early hypoxia on the expression of DV and skeletogenic regulatory genes, hypoxia applied after the early blastula stage does not affect the expression of these genes and results with overall normal skeletogenic patterning (Fig. 6). This resilience of the DV GRN to late hypoxia can be explained by the structure of the GRN, which includes positive- and negative-feedback loops that restrain Nodal activity and robustly maintain the DV axis polarity (Fig. 7A,B; Duboc et al., 2008; Nam et al., 2007; Range et al., 2007). At the early blastula stage, *nodal* expression is maintained by the Nodal pathway through the transcription factors SMAD2 and SMAD3 (Nam et al., 2007; Range et al., 2007), and *nodal* spatial expression is restricted by its antagonist, Lefty, which is also activated by the Nodal pathway (Fig. 7A; Duboc et al., 2008). Once the spatial domain of Nodal activity is established and stabilized by the Nodal-Lefty feedback loops, it is not disrupted by hypoxic conditions, indicating that the redox state is no longer a factor in *nodal* regulation at this stage (Fig. 6H). Nodal spatial activity defines the domain of BMP activity through the Nodal-BMP2/4-Chordin incoherent feedforward loop, which restricts VEGF activity that leads to normal skeletogenic patterning, in late hypoxia embryos (Figs 6 and 7A). This structure of the DV GRN could also underlie the relative restriction of *nodal* expression at the gastrula stage compared with its broad expression at the blastula stage (Fig. 4H,K) and the partial recovery of skeletal patterning in the pluteus stage in early hypoxia (Fig. 4G). Overall, it seems that the structure of the DV GRN enables it to partially recover the effect of early hypoxia at later developmental stages and makes it resilient to hypoxia applied after the DV axis had formed.

Although the effect of the hypoxic conditions on the regulatory cascade downstream of Nodal signaling is quite clear from our findings, the cause of nodal expansion in early hypoxia requires further investigation. Early hypoxia was shown to expand the expression of the HIF1α protein that is normally localized at the dorsal side (Chang et al., 2017). HIF1α transiently represses early *nodal* expression (Chang et al., 2017), yet HIF1α expansion does not restrict *nodal* expression that expands dorsally in early hypoxia (Fig. 4). Hypoxia was shown to increase ROS signaling in smooth muscle cells and endothelial cells (Chi et al., 2010; Desireddi et al., 2010). If hypoxia increases the ROS levels at the already oxidizing side (Fig. 1), this could underlie the expansion of *nodal* expression in early hypoxia. Yet, a previous study had shown that hypoxia decreases the levels of ROS (H_2_O_2_) in sea urchin embryos (Coluccio et al., 2011) and other studies show a complex relationship between hypoxia and ROS levels (Fuhrmann and Brune, 2017; Tafani et al., 2016). Thus, the regulatory mechanism that leads to *nodal* expansion under early hypoxic conditions is yet to be found.

Our findings illuminate some similarities between the GRNs that pattern the DV axis and skeletogenesis in the sea urchin embryo, and the upstream regulation of vertebrate vascularization (Lee et al., 2009; Ushio-Fukai and Nakamura, 2008). Hypoxia and redox gradients that regulate DV axis formation and skeletal patterning in the sea urchin embryo have been shown to induce angiogenesis in vertebrates during normal development and in cancer (Chi et al., 2010; Potente et al., 2011). The regulatory interactions between BMP and VEGF that are essential for sea urchin skeletal patterning, also control vertebrate vascularization; however, they are rather complex: BMP activates VEGF and induces vascularization in some tissues, while it represses VEGF in other tissues (Bai et al., 2013; Dyer et al., 2014; García de Vinuesa et al., 2016; He and Chen, 2005; Wiley et al., 2011). The Nodal pathway does not participate in hypoxia-induced vascularization during normal development in vertebrates; however, in various cancer cells, hypoxia drives Nodal expression, which then promotes *VEGF* expression and angiogenesis (Fig. 7C; Hueng et al., 2011; Quail et al., 2011, 2012). The transcription factor HIF1α is a key activator of VEGF expression and angiogenesis in vertebrates, but the sea urchin HIF1α does not regulate VEGF signaling during normal development (Figs 3 and 7). Overall, regulatory interactions between Nodal, BMP, HIF1 and VEGF pathways, and their modulation by hypoxic conditions are observed both during DV and skeletal patterning in the sea urchin embryo and in vertebrate vascularization, but there are some apparent differences in the linkages. The participation of these common pathways together with the similarity between the skeletogenic and the vascularization GRNs (Morgulis et al., 2019; Oliveri et al., 2008) might indicate that these upstream patterning programs diverged from a common ancestral GRN; yet we cannot exclude convergent evolution at this stage.

Our findings have implications on the effect of ocean deoxygenation on embryos that use hypoxia and redox signaling in their development, yet the major differences between lab experiments and field conditions should be considered. Our analyses and previous studies suggest that the use of hypoxia and redox gradients makes the sea urchin GRNs highly sensitive to acute hypoxia applied in its early developmental stages, but the GRNs are less sensitive to hypoxia applied after the establishment of the DV axis. Yet, hypoxia events in the ocean and in the coastal zones can last for weeks and their lethal effect is observed for months afterwards (Altieri et al., 2017; Hughes et al., 2020). So even if the sea urchin embryos can survive 16 h of hypoxia, they will probably die in longer periods of low oxygen. Furthermore, in other organisms, ROS and hypoxia signaling regulate multiple developmental processes and, in some cases, these processes last throughout embryogenesis, which could make the embryos of these organisms even more sensitive to hypoxia than sea urchin embryos (Breus and Dickmeis, 2020; Coffman and Su, 2019; Cordeiro and Tanaka, 2020). In line with these alarming observations, lab experiments can show distinct and even opposing trends compared with experiments that are carried out in the field, owing to the increased and unexpected complexity of natural sites (Foo et al., 2020). Therefore, further hypoxia studies guided by environmental changes need to be carried out in the field, to elucidate the sensitivity and resilience of the molecular response to hypoxia in marine embryos in their natural habitat.

## MATERIALS AND METHODS

### Animals and embryo cultures

Adult *P. lividus* sea urchins were purchased from the Institute of Oceanographic and Limnological Research (IOLR) in Eilat, Israel. Eggs and sperm were obtained by injection 0.5 M KCl solution to adult sea urchins. Embryos were cultured in artificial seawater (ASW) at 18°C.

### Microinjection, RNA extraction and reverse transcription

The design and preparation of novel morpholino (MO) was carried out in Genetools (http://www.gene-tools.com). Translation of *HIF1α* was blocked by the microinjection of 400-700 µM *HIF1α*-MO (5′-GGTCGCCATAATCAGTCTCTGTTTC-3′) into sea urchin eggs. Translation of *BMP2/4* was blocked by the microinjection of 400-600 µM *BMP2/4* MO (5′-GACCCAGTTTGAGGTGGTAACCAT-3′); this MO has been characterized in previous studies (Duboc et al., 2004). The control MO is a random commercial MO that does not have any effect on embryo development, along with 1 µg/ml rhodamine dextran (D3329, Molecular Probes) and 0.12 M KCl. Total RNA was extracted from injected sea urchin embryos (≥120 injected embryos) using RNeasy Micro Kit (Qiagen, 74004) according to the kit protocol using DNase treatment from RNease-Free DNase Set (Qiagen, 79254). Elution was carried out in 16.5 µl nuclease-free ultra-pure water. Extracted RNAs were then reverse transcribed into cDNA by using SuperScript II Reverse Transcriptase (Thermo Fisher Scientific, 18064022) (10 min at 25°C, 2 h at 25°C and 85°C for 5 min).

### Quantitative-PCR (qPCR) analysis

qPCR was performed using the CFX384 Touch Real-Time PCR Detection System (BioRad, 1855485). Reactions were carried out in 10 μl volume including: 5 µl SYBR BioRad IQ SYBR Green Supermix (1725125), 2.5 μl of 1.2 μM forward and reverse gene specific primers, and 2.5 μl of cDNA (qPCR primers used in this study are listed in Table S1). Each cDNA sample was run in triplicate, for every candidate gene, ubiquitin was used as an internal control. The reactions thermal profile was: 95°C for 3 min followed by 40 amplification cycles of 95°C for 10 s and 55°C for 30 s. Dissociation analysis was performed at the end of each reaction to confirm the amplification specificity. Primer sets for all tested genes were designed using Primer3Plus (http://www.bioinformatics.nl/cgi-bin/primer3plus/primer3plus.cgi/). Results are presented as the mean±s.e.m. of at least two biological replicates. The comparison with an internal standard (ubiquitin) was carried out in order to determine the expression level of the gene, and the change in the expression levels were measured in comparison with the expression level of the gene in control MO.

### Hypoxia treatment

ASW was treated with 99.5% nitrogen (N_2_) and 0.5% oxygen (O_2_) to decrease the oxygen solubility in ASW until the dissolved O_2_ level was 0.4-0.5 mg/l, creating hypoxic ASW. Embryos were transferred into petri dish that contains the hypoxic ASW, then the dishes were incubated in a hypoxia chamber at 18°C. The hypoxia chamber is a sealed box that receives a constant flow of 99.5% N_2_ and 0.5% O_2_. To distinguish between the direct effect that hypoxic conditions might have on skeletogenesis and its effect on DV patterning, we studied the skeletogenic phenotypes of hypoxia applied immediately after fertilization and up to blastula stage (early hypoxic condition), and the effect of hypoxia applied from early blastula stage to gastrula stage (late hypoxic condition). In early hypoxia treatment, the eggs were fertilized, their fertilization envelope was immediately removed and the zygotes were incubated in the hypoxia chamber for 16 h. In late hypoxia treatment, the eggs were fertilized and the embryos were cultured under normoxic conditions for 10 h until the early blastula stage. The embryos were then transferred into the hypoxia chamber and incubated in hypoxic conditions for 16 h. After 16 h in hypoxic conditions, the embryos were removed from the hypoxia chamber and cultured in normoxic conditions until the pluteus stage.

### Probe design and WMISH procedure

WMISH probe preparation and WMISH procedure were performed as described previously (Morgulis et al., 2019). Primer list is provided in Table S2.

### Removal of fertilization envelope

To perform WMISH on sea urchin embryos at early blastula stage, the fertilization envelope (FE) was removed. Fertilized eggs were incubated in the presence of paraminobenzoic acid (PABA, A6928, Sigma) and amino triazole (ATA, A8056 Sigma) (2 mM each at final concentration) to soften the FE. After microscope visualization of the FE, it was removed by passing the zygotes through a 75 µm mesh four times. Next, the embryos were washed three times with ASW and grown until the indicated collection time points.

### Immunostaining

Immunostaining of pSMAD1/5/8 was carried out similarly to previously published methods (Lapraz et al., 2009) with minor modifications. Embryos were fixed in 4% paraformaldehyde, 33 mM maleic acid buffer (pH 7) and 166 mM NaCl, for 10 min at room temperature, then exposed to methanol for 1 min. Embryos were washed four times with PBST, then incubated for 1 h in blocking solution (PBST and 4% sheep serum), followed by incubation with primary antibody against pSMAD1/5/8 (Cell Signaling Technology, 9511; 1:200) in blocking solution, overnight at 4°C. Embryos were then washed four times in PBST, then the secondary antibody was added to the embryos (peroxidase-conjugated AffiniPure goat anti-rabbit IgG, 111-035-003; 1:200) in blocking solution and incubated for 1 h in room temperature, followed by four washes with PBST. The storage solution (PBST in 50% glycerol) was kept at 4°C.

### Imaging

All images presented in this study were generated on a Zeiss Axio Imager M2.
